# Three-Dimensional Simulation of Particle-Induced Mode Splitting in Large Toroidal Microresonators

**DOI:** 10.3390/s20185420

**Published:** 2020-09-22

**Authors:** Lei Chen, Cheng Li, Yumin Liu, Judith Su, Euan McLeod

**Affiliations:** 1 International Collaborative Laboratory of 2D Materials for Optoelectronics Science and Technology of Ministry of Education, Institute of Microscale Optoelectronics, Shenzhen University, Shenzhen 518060, China; leichen@szu.edu.cn; 2 College of Optical Sciences, University of Arizona, Tucson, AZ 85721, USA; chengli@optics.arizona.edu (C.L.); judy@optics.arizona.edu (J.S.); 3 State Key Laboratory of Information Photonics and Optical Communications, Beijing University of Posts and Telecommunications, Beijing 100876, China; 4 Department of Biomedical Engineering, University of Arizona, Tucson, AZ 85721, USA

**Keywords:** whispering-gallery mode, mode splitting, particle sizing, finite-element method, large resonators

## Abstract

Whispering gallery mode resonators such as silica microtoroids can be used as sensitive biochemical sensors. One sensing modality is mode-splitting, where the binding of individual targets to the resonator breaks the degeneracy between clockwise and counter-clockwise resonant modes. Compared to other sensing modalities, mode-splitting is attractive because the signal shift is theoretically insensitive to the polar coordinate where the target binds. However, this theory relies on several assumptions, and previous experimental and numerical results have shown some discrepancies with analytical theory. More accurate numerical modeling techniques could help to elucidate the underlying physics, but efficient 3D electromagnetic finite-element method simulations of large microtoroid (diameter ~90 µm) and their resonance features have previously been intractable. In addition, applications of mode-splitting often involve bacteria or viruses, which are too large to be accurately described by the existing analytical dipole approximation theory. A numerical simulation approach could accurately explain mode splitting induced by these larger particles. Here, we simulate mode-splitting in a large microtoroid using a beam envelope method with periodic boundary conditions in a wedge-shaped domain. We show that particle sizing is accurate to within 11% for radii a<λ/7, where the dipole approximation is valid. Polarizability calculations need only be based on the background media and need not consider the microtoroid material. This modeling approach can be applied to other sizes and shapes of microresonators in the future.

## 1. Introduction

Whispering-gallery mode (WGM) optical microresonators possess ultrahigh quality (Q) factor and subwavelength mode volume, enabling them to probe nanoparticles and their properties [1,2,3]. Local perturbations inside the evanescent field can induce spectral changes, categorized into the spectral shift of the WGM’s resonance frequency, the broadening of the WGM’s linewidth, and the splitting of degenerate WGMs. There are advantages and disadvantages to each of these sensing mechanisms. Sensing based on resonance frequency shifts is highly sensitive and commonly used in the determination of analyte concentrations, as well as single particle detection [4]. However, the spectral shift depends on the location at which the particle binds to the resonator. In addition, for sensing low concentrations of molecules, which is needed for early disease detection, the resonance shift is influenced by ambient conditions like temperature and can increase the experimental noise floor. Linewidth broadening sensing relies on the perturbation inducing energy losses through absorption or scattering. This method is less sensitive to the environment, but the optical properties and size of the perturbing particle cannot be uniquely determined by the spectral measurement alone. Finally, sensing based on mode-splitting makes use of both resonance frequency and linewidth changes of pairs of degenerate WGMs, i.e., the clockwise (CW) and the counter-clockwise (CCW) modes that share the same resonance frequency and field distribution in the unperturbed resonator but travel in opposite directions [5,6,7,8,9,10,11,12,13,14]. Unlike the other modalities, mode-splitting can size particles based on spectral features alone and is robust to environmental temperature and pressure variations, which do not induce mode-splitting.

Mode-splitting in microtoroids has been experimentally verified to size nanoparticles as small as 30 nm in radius [5]. In theory, smaller particles could be sized if composed of a more polarizable material or if the microcavity had a higher intrinsic Q. Theoretical predictions indicate that sizing particles as small as ~10 nm in radius should be within reach [5,6,7,15]. Particle-induced mode splitting is most often modeled using a Weisskopf–Wigner semi-QED treatment [16]. The result of this purely analytical model is that the polarizability of a binding particle can be deduced from the ratio of the broadening of one of the modes to the splitting between the two modes [5]. Unlike shift-based sensing, this ratio is insensitive to the local evanescent field strength where the particle binds, making it independent of binding location. If the particle material is known, particle size can be deduced from its polarizability. However, this analytical approach is based on two assumptions that may not always be true: (1) the dipole assumption, which incurs significant error when the particle size approaches λ/10 [17], and (2) the assumption that the scattering and polarizability of a particle sitting on the surface of the microresonator are the same as in a homogeneous medium. Some experimental and numerical studies have found discrepancies between the deduced and true particle sizes based on this analytical theory, perhaps due to the failure of one or both of these assumptions [6,13]. Theoretical predictions can be further complicated by confusions related to the various definitions of polarizability, which sometimes include the free-space permittivity [18], an additional factor related to background index [9], and sometimes are just represented by a polarizability volume [6], depending on the author and system of units.

Finite element method models are generally quite accessible and straightforward to implement in microresonator systems. They have been used to simulate mode splitting in small microresonators [11,19]. However, full 3D simulations of resonators larger than a few microns in diameter are intractable due to the demands on computational memory and processing time.

**Figure 1:**
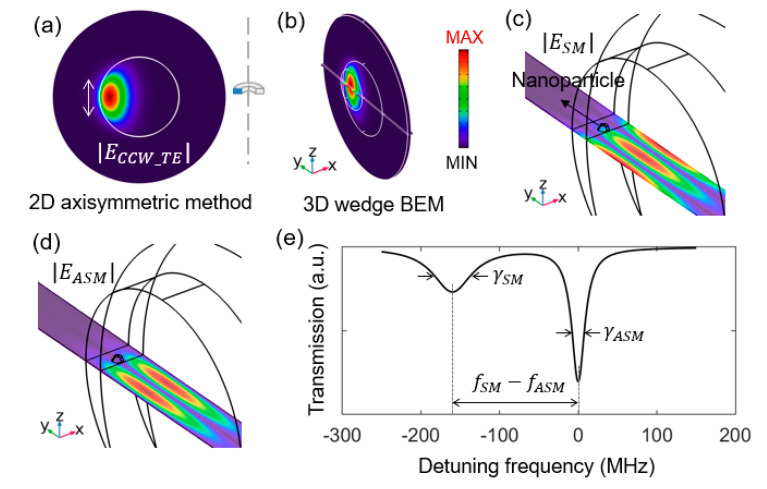
Electric field norm distributions of the traveling transverse-electric (TE) counter-clockwise (CCW) mode inside a bare microtoroid simulated using (**a**) 2D axisymmetric method and (**b**) 3D eigenfrequency. Electric field norm distributions of the (**c**) symmetric (SM) mode and (**d**) antisymmetric (ASM) mode were simulated using a 3D eigenfrequency model. The perturbative polystyrene nanosphere has a radius of 50 nm and is positioned with a 10 nm radial gap between it and the microtoroid equator. (**e**) Theoretically simulated mode splitting transmission spectrum. The SM mode experiences a frequency redshift of 2g and a linewidth broadening Γ, which is quantified by a full width at half maximum linewidth in Hz. The color bar for all electric field norm distributions is given in (**b**).

Experimentally, large microresonators show good spectral resolution and sensitivity because they have ultrahigh Q-factors and compact mode volumes. Furthermore, they have large capture areas that make analyte detection events more likely. These attributes make their accurate simulation a significant goal. In 2013, Kaplan et al. simulated a 3D microtoroid coupled to a gold nanorod and predicted the near-field enhancement for biosensing. The model is reduced to a small wedge using perfect electrical conductor (PEC) boundary conditions [20]. However, mirror boundary conditions such as these cannot be used to simulate mode-splitting because the modes will differ in phase by π/2 (as seen in Figure 1c,d), while PEC boundary conditions force the tangential electric field to be zero at the boundary for all solutions. In 2016, Han simulated a 2D microcylinder coupled to a nanocylinder and predicted WGM frequency shifts in the transmission spectrum [21]. However, this model cannot simulate nanoparticles (spheres, rods, triangles, etc.) and was only used for microresonators like cylinders or rings.

## 2. Modeling Approach

Here, we use a 3D periodic wedge-shaped beam envelope method (BEM) model with periodic boundary conditions to simulate mode-splitting and particle sizing using a 90-µm diameter microtoroid. Advantages of finite element BEM modeling over more common finite difference time domain (FDTD) models include lower memory requirements due to larger mesh sizes enabled by the BEM approach, faster computation times due to modeling in the frequency domain rather than time domain, and more accurate results due to more conformal polygonal mesh cells rather than a fixed rectangular grid. We have previously used a related, but different, Floquet boundary condition method to model coupling of free-space light with traveling waves within the microtoroid [22]. Unlike previous numerical simulations of mode-splitting, our method can handle large, three-dimensional, non-spherical microresonators and recover the eigenfrequencies of the split modes from a single simulation. All simulations are carried out using COMSOL Multiphysics (wave optics module).

When the microtoroid is only weakly perturbed, the splitting of the two degenerate modes is less than their linewidth, and the spectral response is dominated by the shift in resonance. For larger perturbations, the degenerate travelling wave modes split into two standing wave modes. According to the position of the nanoparticle, these modes redistribute themselves as a symmetric (SM) mode and an antisymmetric (ASM) mode [5]. Dipole approximation theory is used to describe the nanoparticles, which is valid when the nanoparticle radius is much less than the free-space wavelength ($a_{0}\ll \lambda$) [23,24]. In this case, the WGM’s evanescent field induces a dipole moment inside the nanosphere proportional to its polarizability:

(1) $$\alpha =3V_{np}\frac{\varepsilon_{np}-\varepsilon_{bg}}{\varepsilon_{np}+2\varepsilon_{bg}}$$

where $V_{np}=4\pi a_{0}^{3}/3$ is the dielectric nanosphere volume, $\varepsilon_{np}$ is the relative permittivity of the nanoparticle, and $\varepsilon_{bg}$ is the relative permittivity of the background medium. This is the most commonly used polarizability definition in the analysis of mode splitting [5,16]. As introduced in [5,16], for the split modes, the coupling rate between the modes is:

(2) $$g=-\frac{\alpha f{\left(r\right)}^{2}\omega_{c}}{2V_{m}}$$

where f(r) is the normalized WGM mode field distribution, $\omega_{c}=2\pi c/\lambda_{c}$ is the angular resonance frequency of the unperturbed cavity mode, and $V_{m}$ is the mode volume. The additional damping rate for the split modes is:

(3) $$\Gamma =\frac{\alpha^{2}f{\left(r\right)}^{2}\omega_{c}^{4}}{6\pi v^{3}V_{m}}$$

where $v=c/\sqrt{\varepsilon_{bg}}$, and c represents the speed of light in vacuum. The values of g and Γ can be recovered from experimental or simulation results (e.g., Figure 1e) regarding the spectral shape: $2g=2\pi \;\left(f_{SM}-f_{ASM}\right),$ and $2\Gamma =2\pi \left(\gamma_{SM}-\gamma_{ASM}\right),$ where the linewidths γ correspond to full width at half-maximum values. In our 3D eigenfrequency simulations, $f_{SM}$ and $f_{ASM}$ are returned as the real part of the eigenfrequencies, while the linewidths are equal to twice the imaginary part of the eigenfrequencies [18]. Computing the ratio of Equation (3) to Equation (2), and solving for the particle volume yields:

(4) $$V_{np}=\frac{-{\left(\lambda_{c}/\sqrt{\varepsilon_{bg}}\right)}^{3}\left(\varepsilon_{np}+2\varepsilon_{bg}\right)}{4\pi^{2}\left(\varepsilon_{np}-\varepsilon_{bg}\right)}\cdot \frac{\Gamma }{2g}$$

The advantages of mode-splitting can be seen from this equation, where the particle size only depends on the particle-WGM interaction quantified by Γ/2g and relative permittivities of the nanoparticle and background medium, and not the local field strength f(r). The value of Γ/2g is therefore independent of the particle location on the surface of the microtoroid.

A 2D axisymmetric eigenfrequency simulation is run to probe the resonance features of the bare microtoroid (see Figure 1a). In the 2D axisymmetric formulation, the angular dependence of the electric field is given by $e^{\pm im\varphi }$, where an integer m=660 is specified to search for a WGM around $\lambda_{c}\approx 633$ nm. The simulated microtoroid has a major (minor) radius of 45 µm (2 µm). The background is $5\lambda_{c}/\sqrt{\varepsilon_{bg}}$ in length in the radial direction beyond the edge of the toroid. The simulation domain involves the microtoroid and the background medium. Scattering boundary conditions are used for the exterior boundaries of the simulation domain. An imaginary part is added to the refractive index of the silica ($\sqrt{\varepsilon_{SiO_{2}}}=1.45+i10^{-8}$) to maintain an intrinsic Q of $7.4\times 10^{7}$. Toroids with reduced Q due to imperfect fabrication could be simulated by increasing the imaginary part of the refractive index in the model.

To run efficiently, the 3D BEM requires an approximate phase specification [25]; in this case, the angular dependence of the electric field $e^{-i\Phi }$ needs to be given a priori as Φ=−m atan2(y,x) (=+m atan2(y,x)) for the TE CCW (CW) mode. Different from a 2D cross-section, the microtoroid is reduced to a small 3D wedge with an xy plane azimuthal angle of $\theta_{w}=2\pi /m\;$(see Figure 1b). The wedge is flanked by periodic boundary conditions. The background size and the scattering boundary are the same as in the 2D axisymmetric simulation. Within the cross-section, we define a circular domain of radius 0.5 µm enclosing the WGM mode and most of its evanescent field for mesh refinement (see Figure 1b) with cells smaller than $\lambda_{c}/8\sqrt{\varepsilon_{SiO_{2}}}$, while leaving the remaining domains at a lower-density mesh (cells < $\lambda_{c}/6\sqrt{\varepsilon_{SiO_{2}}}$). The number of nanoparticles that can be simulated depends on the size of the refined mesh domain. Free triangular elements and a copied mesh are used for the wedge faces, and a swept mesh for azimuthal direction. In the absence of particles, simulation results of the 2D axisymmetric and 3D periodic BEM methods give the same resonance wavelength and linewidth for the unperturbed WGM, validating our 3D periodic BEM approach. In the presence of a particle, WGM mode-splitting induced by the particle is simulated using the 3D periodic BEM.

## 3. Results and Discussions

**Figure 2:**
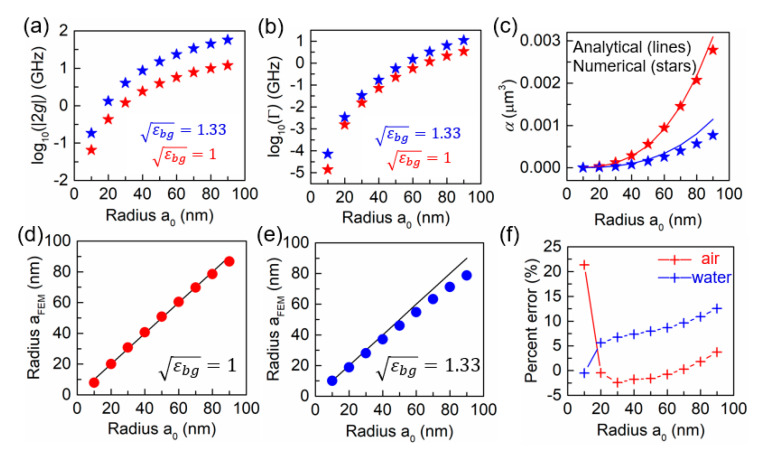
Three-dimensional eigenfrequency simulation results of (**a**) the splitting frequency |2g| and (**b**) linewidth broadening Γ versus radius $a_{0}$ in terms of two different background media: air with $\sqrt{\varepsilon_{bg}}=1$ and water with $\sqrt{\varepsilon_{bg}}=1.33$. (**c**) Nanosphere polarizability versus radius $a_{0}$. Solid lines denote the analytical calculation using Equation (1) and stars denote numerical results derived from $\Gamma /2g=-\alpha \omega_{c}{}^{3}{\sqrt{\varepsilon_{bg}}}^{3}/\left(6\pi c^{3}\right)$. (**d**,**e**) Particle radius $a_{\mathrm{FEM}}$ derived from Equation (4). Solid lines indicate the true radius $a_{0}$. (**f**) Percent error of the sizing results calculated by $100\times \left(a_{0}-a_{\mathrm{FEM}}\right)/a_{0}$.

The following are the 3D simulation results for mode splitting and nanosphere sizing. Mode splitting occurs when the microtoroid is locally perturbed by a polystyrene nanosphere with refractive index of $\sqrt{\varepsilon_{np}}=1.59$. In the simulation, the nanosphere is placed at the equator of the microtoroid. Figure 2a,b show the splitting frequency 2g and the linewidth broadening Γ versus radius $a_{0}$ in two different background media: air with $\sqrt{\varepsilon_{bg}}=1$ and water with $\sqrt{\varepsilon_{bg}}=1.33$. Based on simulations of particles of slightly varying refractive index, we find that for this particular system, the sensitivity of the mode splitting, defined as the magnitude of splitting per particle refractive index unit (RIU) per particle volume is $2.16\times 10^{-5}$ nm RIU^−1^ nm^−3^ in water and $2.97\times 10^{-6}$ nm RIU^−1^ nm^−3^ in air. The condition for mode splitting to be resolvable is $\left|2g\right|>\Gamma +\Gamma_{0}$, where $\Gamma_{0}$ is the intrinsic linewidth of the unperturbed WGM [6] For imperfect toroids with large $\Gamma_{0}$, the splitting may not be resolvable. On the other hand, if the intrinsic linewidth is small, a simplified condition is |2g|>Γ [16]. In our system, the large microtoroid has an ultrahigh Q-factor with $\Gamma_{0}$ < 0.01 GHz, so that both conditions hold for nanospheres with radius of 10–100 nm. The SM mode experiences a resonance wavelength redshift due to $\varepsilon_{np}>\varepsilon_{bg}$. Figure 2c shows the polarizability α of the nanosphere in the presence of mode splitting. The solid lines denote the α calculated from Equation (1) and the volume of a sphere, while the stars denote the particle sizes calculated using Equation (4) and the results of the numerical simulations. The relative polarizability is greater in an optically thinner medium (i.e., air). Figure 2d,e show particle sizing using the 3D wedge BEM simulation approach. Figure 2f shows percent error in particle size for both cases. The error is within an acceptable range of 0.25–3.7% for particles with $20\leq a_{0}\leq 90$ nm in air, and 5.6–9.6% for particles with $20\leq a_{0}\leq 70$ nm in water. These low errors demonstrate the consistency between the numerical and analytical mode-splitting models for particles of theses sizes, providing validation of the models. In the case of water background, sizing error becomes more distinct as the radius increases, presumably due to the decreasing accuracy of the dipole approximation. The dipole approximation fails in water for smaller particles than in air because the effective wavelength in water is shorter than in air. The relatively large percent error for 10 nm radius particles in air is due to the small magnitude of both |2g| and Γ as shown in Figure 2. We note that although the percent error in this case is ~20%, the absolute sizing error remains low.

**Figure 3:**
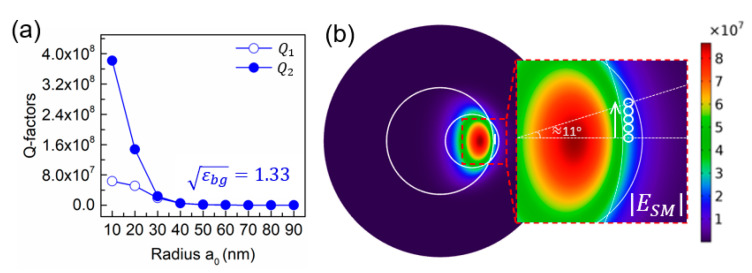
(**a**) Q-factors of the SM mode versus the radius $a_{0}$. (**b**) Diagram of the five microtoroid-particle binding cases where the particle lands with five different polar angles moving away from the energy maximum of the whispering-gallery mode (WGM). The electric field units are arbitrary.

To compare the contributions to Q-factor from particle-induced losses with the intrinsic microtoroid Q-factor, we calculate the Q-factors of the coupled WGMs using two methods, both resulting from 3D eigenfrequency simulations: $Q_{1}=Re\{f_{\mathrm{res}}\}/(2Im\{f_{\mathrm{res}}\})$ (hollow blue dots) and $Q_{2}=2\pi Re\left\{f_{\mathrm{res}}\right\}W/P$ (solid blue dots), where $f_{\mathrm{res}}$ is the complex eigenfrequency of the symmetric mode, W is the intracavity energy, and $P=P_{\mathrm{abs}}+P_{\mathrm{rad}}$ the total loss dissipated by the particle [22]. In the simulations, W is a volume integral of the time-average energy density over the microtoroid domain, $P_{\mathrm{abs}}$ is the volume integral of the total power dissipation density over the nanosphere domain, and $P_{\mathrm{rad}}$ is the surface integral of the time-average power flow over all outer surfaces in the radial direction. The first model, $Q_{1}$, is an accurate model of the net Q-factor, accounting for both intrinsic and particle-induced losses, whereas the second model, $Q_{2}$, neglects the contribution from the intrinsic loss in the microtoroid. As shown in Figure 3a, particle-induced losses dominate for particle radii $a_{0}\geq 30$ nm.

In theory, the mode splitting Γ/2g is independent of the particle location on the surface of the microtoroid. As given in Table 1, we simulate five microtoroid-particle binding cases where the particle lands with five different polar angles (see Figure 3b). We attribute the small fluctuations in sizing error as a function of polar angle to slight differences in the finite element mesh at those locations. For very large distances from the equatorial plane, mode splitting may become unresolvable due to decreased electromagnetic interaction between the particle and the WGM. These locations are currently outside of our region of high mesh density, but could be accurately computed using a new model with different zones of mesh refinement.

## 4. Conclusions

In summary, we used a computationally efficient 3D simulation method to simulate mode splitting induced by a nanoparticle positioned in the evanescent field of a large microtoroid. Q-factors can be recovered from a single simulation run. We find excellent agreement between analytical theory and simulation for nanoparticles of radius up to 70 nm in water and up to 90 nm in air, which both correspond to $\lambda_{\mathrm{eff}}/7$ and the breakdown of the dipole approximation. This agreement validates the approach for calculating polarizability based on a single homogeneous background medium, rather than considering both the surrounding fluid and silica toroid as background media in calculating the particle polarizability. We also confirm the robustness of the sizing approach to binding at different polar coordinates. Our method can simulate mode-splitting induced by particles beyond the dipole approximation, i.e., beyond the limits of the existing analytical theory. In the future, the model could be improved to handle more nanoparticles in more locations through adaptive meshing and mesh refinement. As a computationally fast and versatile approach, our model has the potential to guide future experiments using microcavities. As one example, our approach could be used to directly model the impact of isolated surface defects on the performance of microtoroids in other applications, as these defects are optically equivalent to the analyte nanoparticles interacting with the sensor that we simulated here.

## Figures and Tables

**Table 1:** Particle sizing for nanospheres on the rim of the microtoroid with different angles in yz-plane. The simulated nanosphere has a radius of $a_{0}=50$ nm. The percent errors in size are 0.86–7.82% and 7.42–10.38% in terms of background air with $\sqrt{\varepsilon_{bg}}=1$ and water with $\sqrt{\varepsilon_{bg}}=1.33$, respectively.

Angle above Equator	$a_{FEM}\;\left(nm\right),\sqrt{\varepsilon_{bg}}=1$	$a_{FEM}\;\left(nm\right),\sqrt{\varepsilon_{bg}}=1.33$
0°	50.78	46.01
2.75°	51.06	46.09
5.5°	53.91	46.29
8.25°	50.43	45.13
11°	52.17	44.81
